# Understanding low uptake of contraceptives in resource-limited settings: a mixed-methods study in rural Burundi

**DOI:** 10.1186/s12913-017-2144-0

**Published:** 2017-03-15

**Authors:** M. Ndayizigiye, M. C. Smith Fawzi, C. Thompson Lively, N.C. Ware

**Affiliations:** 1 Partners In Health-Lesotho, House No. 233, Maseru West Cnr Lancers and Caldwell Rd., Maseru, Lesotho; 2 Department of Global Health and Social Medicine, Harvard Medical School, 641 Huntington Ave., Boston, MA USA

**Keywords:** Fertility, Family Planning, Contraceptives, Africa, Rural Burundi

## Abstract

**Background:**

Family planning can reduce deaths, improve health, and facilitate economic development in resource-limited settings. Yet, modern contraceptive methods are often underused. This mixed-methods study, conducted in rural Burundi, sought to explain low uptake of contraceptives by identifying utilization barriers. Results may inform development of family planning interventions in Burundi and elsewhere.

**Methods:**

We investigated uptake of contraceptives among women of reproductive age in two rural districts of Burundi, using an explanatory sequential, mixed-methods research design. We first assessed availability and utilization rates of modern contraceptives through a facility-based survey in 39 health clinics. Barriers to uptake of contraceptives were then explored through qualitative interviews (*N* = 10) and focus groups (*N* = 7).

**Results:**

Contraceptives were generally available in the 39 clinics studied, yet uptake of family planning averaged only 2.96%. Greater uptake was positively associated with the number of health professionals engaged and trained in family planning service provision, and with the number of different types of contraceptives available. Four uptake barriers were identified: (1) lack of providers to administer contraception, (2) lack of fit between available and preferred contraceptive methods, (3) a climate of fear surrounding contraceptive use, and (4) provider refusal to offer family planning services.

**Conclusions:**

Where resources are scarce, availability of modern contraceptives alone will likely not ensure uptake. Interventions addressing multiple uptake barriers simultaneously have the greatest chance of success. In rural Burundi, examples are community distribution of contraceptive methods, public information campaigns, improved training for health professionals and community health workers, and strengthening of the health infrastructure.

**Electronic supplementary material:**

The online version of this article (doi:10.1186/s12913-017-2144-0) contains supplementary material, which is available to authorized users.

## Background

High fertility in developing countries persists, despite a global decline in birth rate. The average number of children per woman in the world has decreased from 6.55 to 4.53 over the last 50 years [1]. The world’s population grows each year by approximately 80 million people. Nearly all of this growth is concentrated in developing nations [2]. In sub-Saharan Africa, for example, the total fertility rate was reported to be 5.39 children per woman in 2012 [1].

There are important negative consequences for health related to high fertility, impacting maternal and child morbidity and mortality, as well as economic development. By increasing the number of births, high fertility also increases the number of times a woman is exposed to the risks of child bearing, e.g. unsafe abortions, iron deficiency anemia, and/or maternal death from hemorrhage or other complications [3–5]. High fertility leads to large families, potentially resulting in food insecurity. Child malnutrition and mortality have been found to be positively associated with fertility rate, family size, and poverty across all the world’s regions [6].

Family planning can reduce fertility and maternal mortality by preventing high risk births and reducing abortions [7, 8]. It can also increase female life expectancy [9]. Smaller, planned families advance economic growth. Small families mean reduced child care, freeing time for participation in paid labor and increasing income per capita [2–4, 10–12].

Despite the benefits of family planning, contraceptives are underutilized in many parts of the developing world [13]. Women in the highest wealth quintile, on average, have the highest level of contraceptive use. Rural women are less likely than urban women to use a modern contraceptive method for a number of reasons, including poor quality of family planning services and limited choice of contraceptive methods [13–15].

Efforts to improve health and quality of life by increasing uptake of family planning in any location must start with an understanding of the problem. Rural Burundi in particular is a setting with high fertility and low utilization of contraceptive methods— understanding the factors that influence the use of family planning services in this context can inform strategies for improving access in this resource-limited setting. A number of questions should be addressed to understand utilization of family planning in this context. What are the barriers to modern contraceptive use? How can these barriers be reduced or overcome? To address these questions, we carried out a mixed-methods study of contraceptive uptake among women in rural Burundi.

## Methods

### Study setting: Burundi

**Fig. 1:**
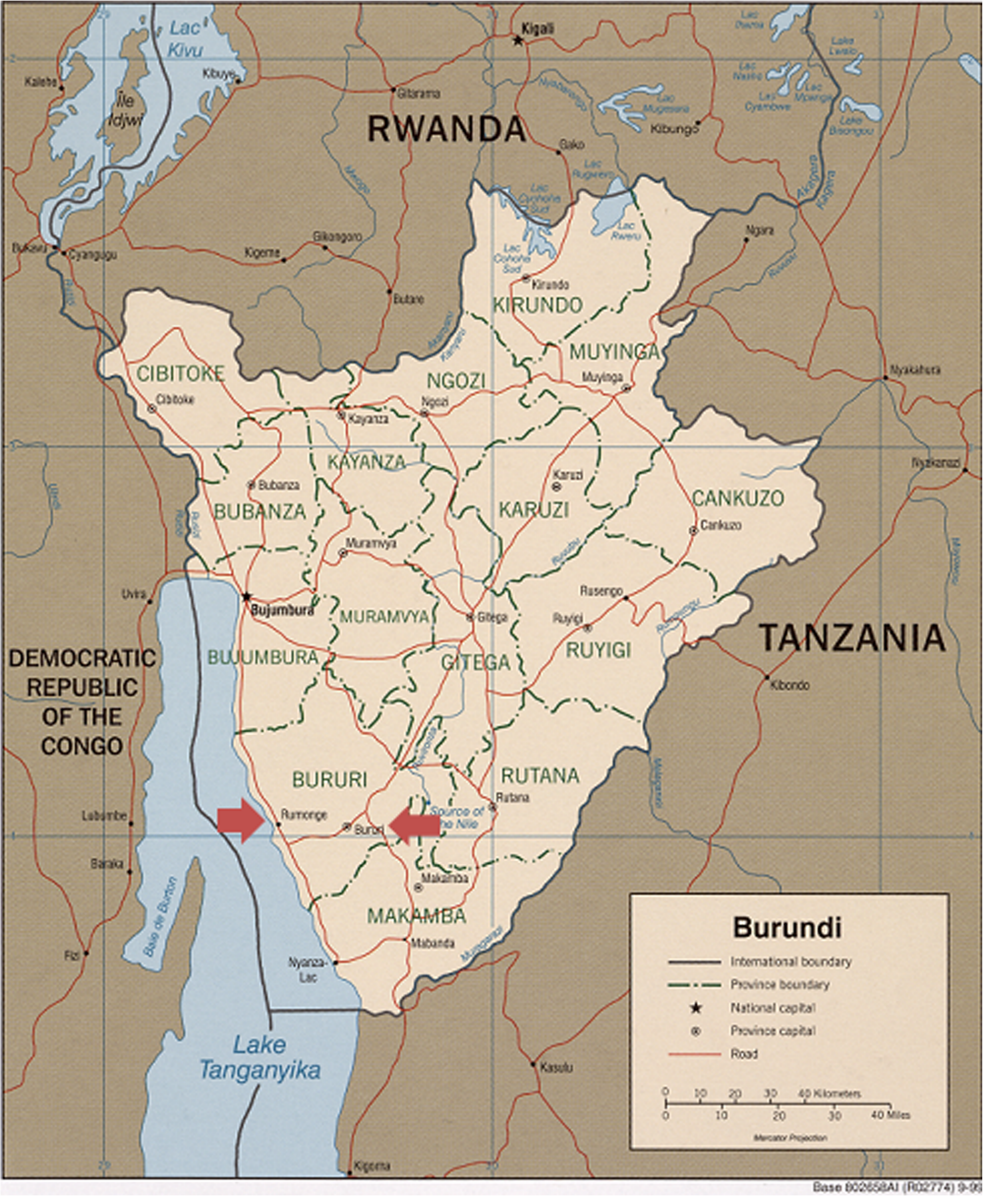
Map of Burundi showing the study locations. This a copyrighted map by the U.S. Central Intelligence Agency, Permission was granted for reprinting the map

Burundi is a small country located in the Great Lakes region of East Africa (Population: 9,233,000) (Fig. 1) [1, 16]. It is a country marked by extreme poverty. Eighty-one percent of Burundians live on less than $1.25 per day [17]. Poverty in Burundi can be traced to several factors.

Most Burundians rely on subsistence farming for their livelihood. The population is dense, and land relatively scarce, leading to overuse of the farmland available. Over-farming, in turn, leads to erosion and soil degradation. In addition, agriculture is hampered by frequent drought, scarcity of tools and technology, and limited incentives and support for improvement of farming techniques. Thus, cash income from farming is low, and few other opportunities for generating income are available.

Rural Burundian communities often lack access to basic services. Safe drinking water, health care, and opportunities for education are all in short supply. Limited access to formal education leads to high illiteracy rates [18].

High fertility also contributes to poverty in Burundi. At 6.4 children per woman, Burundi has the third highest fertility rate in the world [19]. The population grows by 2.4% every year [20]. Poverty in Burundi is exacerbated by recurrent civil wars and political instability. As a result, the population is mobile, reinforcing low productivity [18]*.*

This study was conducted in Rumonge and Bururi rural health districts, located in the southwestern part of Burundi (Fig. 1) [16]. Health districts reflect Burundi’s decentralized public health service system, in which each district is led by a medical director. Each health district has a hospital that serves as a referral hospital for all the local health centers in that region. These two districts were selected for their dense populations. They are among the most densely populated rural regions in Burundi, having accommodated a large number of Burundians repatriating from neighboring countries after the 1972 and 1993–2005 civil wars.

### Mixed-methods study design

**Fig. 2:**
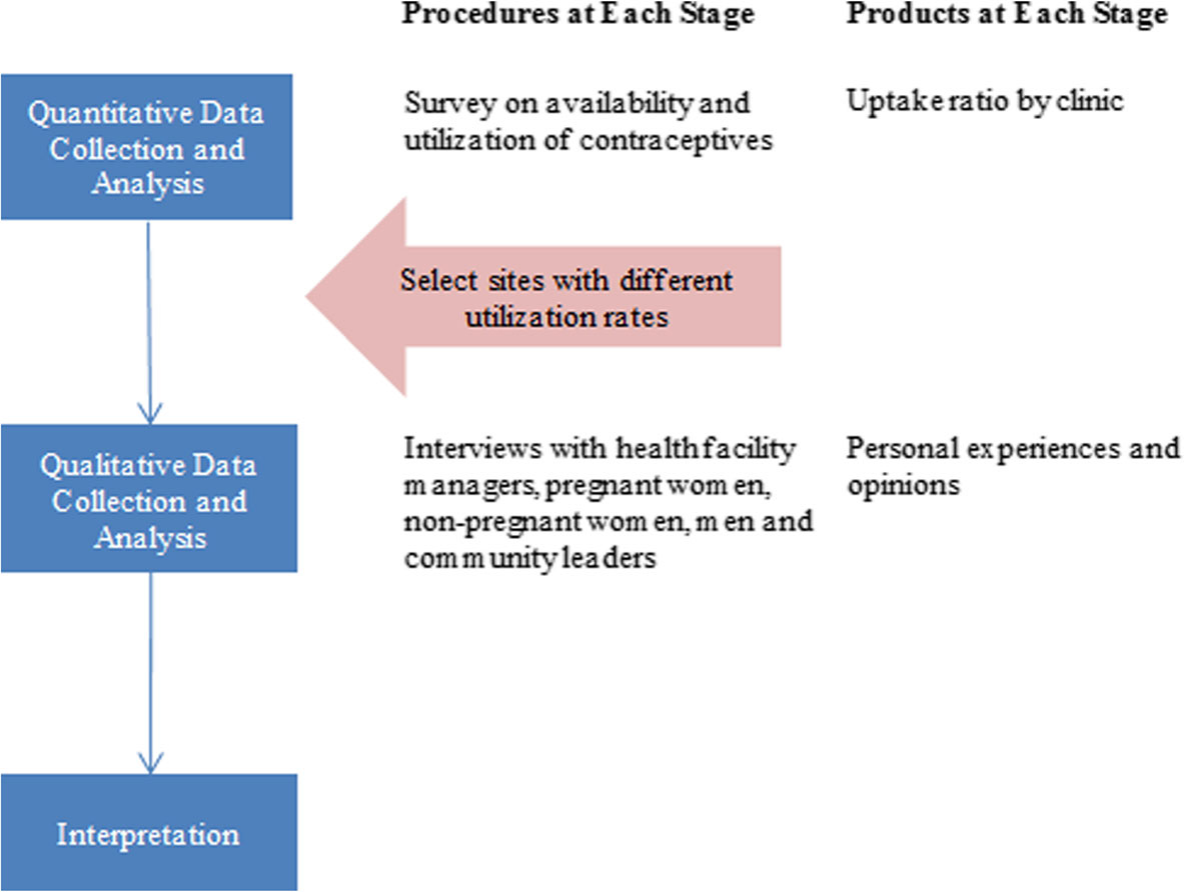
Explanatory sequential design of study of low contraceptive uptake in rural Burundi

This mixed-methods study used an explanatory sequential design. In an explanatory sequential design, quantitative data are collected first; qualitative data are then gathered to interpret quantitative results [21]. In this study, a cross-sectional health facility survey of availability and utilization of contraception was followed by qualitative interviews and focus groups targeting barriers to contraceptive use, as shown in Fig. 2. Thus the study drew upon two kinds of data: (1) secondary, administrative data for the quantitative (survey) component; and primary data (collected specifically for this investigation) for the qualitative component. The quantitative statistical results are presented first, followed by inductively derived, qualitative categories aimed at explaining the quantitative results [21–23].

### The research team

This research was carried out by a multidisciplinary research team that included a general physician and native of Burundi (primary author MN), an epidemiologist (second author MCSF), a medical educator (third author CTL), and a medical anthropologist (senior author NCW). Qualitative and quantitative study data were collected by the primary author in partial fulfillment of the requirements for a graduate degree. Before the study was conducted, the primary author (as a Burundian physician) had extensive experience in the study setting. As medical director of a local clinic, he was known by heads of local political and/or geographic subdivisions (who referred potential study participants), by community health workers, and by facility managers. He was not personally acquainted with individuals who participated in the qualitative component of the study, although they may have recognized him by name and/or reputation in his professional role.

#### Data collection for the quantitative component: survey of health facilities in Rumonge and Bururi districts

All 39 health facilities providing family planning services in Rumonge and Bururi districts were assessed for availability and utilization of modern contraceptives over the 12 months from July 2012 through June 2013 (Additional file 1).

##### Availability of family planning services

Availability of contraceptive services was defined as availability of contraceptives in a given month. We assessed contraceptive availability through a record review. Stock inventory charts provided information on the number of different types of contraceptives available, and on the occurrence and duration (in number of days) of stock-outs. A stock-out was determined if the clinic offered a given contraceptive but it was not available for any part of one month. The following contraceptive methods were examined for availability: (a) depot medroxyprogesterone acetate (DMPA), (b) implant, (c) intra-uterine device (IUD), (d) oral contraceptives, (e) male condoms, (f) female condoms, and (g) emergency contraception (“the morning after pill”).

Availability of service providers was defined as the presence of at least one provider at the facility in a given month. Required monthly reports on the number of providers employed at each facility were used to capture data on provider availability. The following information was collected: (1) number of health professionals working at each facility in general, (2) number of health professionals engaged and trained in family planning service provision, and (3) frequency of provider-led education sessions on family planning.

##### Utilization of family planning services

The source population for collection of data on family planning service utilization was women of reproductive age (between 15–49 years) living in Rumonge and Bururi districts. According to national health statistics, women in this age group represent 23.7% of the total population in these two districts. This translates to a target population of 67,989 women for Rumonge and 30,222 women for Bururi.

The following survey data were collected from monthly reports and clinic logbooks to shed light on utilization of family planning services: (1) number of women coming to the health center for family planning services each month, (2) number of women seeking services who were 15–49 years of age, and (3) number of people in the facility’s catchment area.

Survey data on availability and utilization of family planning services were recorded on structured survey forms by the primary author. The period of survey data collection was August 2013 to December 2013.

#### Sampling, recruitment and data collection for the qualitative component: a study of barriers to family planning use

A qualitative interview study was carried out from December 2013 to January 2014 to explain survey results by identifying and describing barriers to contraceptive use. Sampling, recruitment, and data collection procedures for the qualitative study are described below.

##### Sampling and recruitment for the qualitative study

We used purposeful sampling to ensure that a variety of perspectives on the research topic were systematically represented [24]. Purposeful sampling was implemented at two levels:

1. At the level of facilities, by selecting two facilities with the lowest and two with the highest contraceptive uptake;
2. At the level of individuals, by intentionally selecting study participants from the following groups: (a) pregnant women, (b) non-pregnant women, (c) men, (d) community leaders, and (e) health facility managers.

Participants in the pregnant women group were volunteers. Potential participants in the non-pregnant women, men and community leader groups were referred by the heads of local political and/or geographic subdivisions. The primary author worked with a female research assistant and with community health workers to recruit participants in the qualitative study and collect the data. Pregnant women were approached by the research assistant in conjunction with clinic visits for antenatal care. She described the study and invited individuals who expressed interest to participate. Non-pregnant women and men were recruited through community health workers, who invited them to meet the primary author and the research assistant to discuss participation in the study. Community leaders were identified using the primary author’s knowledge of the area to identify leaders with the greatest influence on health. He personally contacted the community leaders and facility managers and invited them to participate in the study.

##### Data collection for the qualitative study

Two types of qualitative data collection activities were carried out for the qualitative study: (1) focus group discussions (FGD), and (2) individual in-depth interviews. Pregnant women, non-pregnant women, and men took part in FGDs. FGDs were conducted separately for the different participant groups. Facility managers and community leaders took part in individual interviews.

Both FGDs and individual interviews covered the following topics: (a) desired number of children, (b) knowledge of and thoughts about modern contraceptives, (c) preferences for contraceptive methods, (d) influence of sexual partners on ideas about and preferences for contraception, (e) impact of disagreement between partners on contraceptive use, (f) influence of religious beliefs on contraceptive use, (g) influence of the recent 13-year civil war on the desired number of children, and (h) suggestions on how to improve family planning services in the region.

To collect the data, an interview guide was prepared in English (Additional files 2, 3, 4) and then translated into Kirundi, the local language. Slight grammatical modifications were made to the individual interview guide to fit the focus groups. For example, pronouns were changed from the singular to plural in the local language for FGDs. The primary author trained the research assistant in collecting qualitative data – asking open-ended questions and probing – to obtain detailed responses from interviewees. The guide was pre-tested by conducting one FGD with women and one individual interview prior to the start of formal data collection.

The research assistant conducted the FGDs and in-depth individual interviews with women. The primary author conducted the interviews with men, facility managers, and community leaders. The research assistant received daily supervision from the primary author to ensure data quality. Written consent to participate was obtained at the beginning of each FGD or interview.

Individual interviews and focus groups discussions were carried out. Each participant took part in one interview or focus group only. The interviews were conducted in the health care facilities and at a variety of settings in the community where privacy could be assured. They were conducted in Kirundi, the local language, and on average, lasted from 30–60 min. FGDs and individual interviews were audio-recorded with permission. Written notes were taken by the researchers during the interviews to augment the digitally recorded data. The recordings were used to produce complete transcripts of the qualitative data in English.

### Analysis of quantitative and qualitative data

This mixed-methods study generated two datasets: (a) quantitative survey data, and (b) qualitative interview and FGD data.

#### Analysis of survey data

Analysis of survey data began with descriptive statistics, i.e. calculation of frequencies, means, and standard deviations. The unit of observation was the total number of months assessed for all clinics (*N* = 427 months). The contraceptive uptake ratio was calculated using the number of women of reproductive age (15–49 years) in each catchment area of participating health facilities in the two districts as the denominator. The numerator was the number of women enrolled in the family planning program at each clinic per month. Seven clinics were excluded from the analysis because the numbers of people in those catchment areas were not specified. Univariate and multivariate linear regression models were performed with STATA software version SE 12.1 to test the associations between the uptake ratio and (a) availability of contraceptives, (b) number of contraceptives stocked out, (c) number of health providers fully trained in family planning services, and (d) number of health providers engaged in family planning service provision. To utilize linear models, we ensured that each model converged for univariate as well as multivariate models. Factors included in multivariate models were those that were identified *a priori* as predictor variables potentially related to low uptake of contraceptives.

#### Analysis of qualitative data

Following the mixed-methods, explanatory sequential design, qualitative data were analyzed to explain quantitative results. A content analytic approach was used to construct thematic explanatory categories shedding light on reasons for low contraceptive uptake [25, 26]. Study participants did not provide feedback on the explanatory categories. The categories are presented under Results, below.

## Results

Major results from this mixed-methods study are presented in explanatory sequence below. Quantitative findings, reporting on availability and utilization of contraceptives in the research setting, appear as Items A and B. Four barriers to contraceptive uptake, emerging largely from the qualitative data, follow under Item C.

**Fig. 3:**
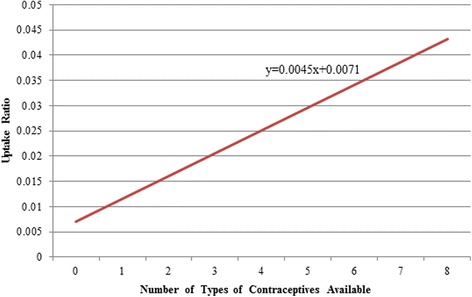
Relationship between availability of contraceptives and uptake ratio

**Fig. 4:**
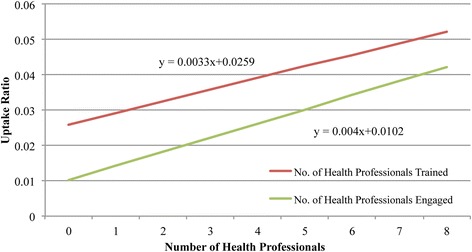
Relationship between uptake and the number of health professionals engaged and trained in family planning

A. **Contraceptives were generally available in the 39 facilities studied.**
  Contraceptives were generally available in all 39 health facilities studied. Two long-term contraceptive methods (DMPA, IUD) and all three short-term methods examined (oral contraceptives, male condoms, female condoms) were available for more than half of the 427-month observation period. DMPA, oral contraceptives, and male condoms were available nearly all of the time. At least one long-term method was available nearly all of the time. Emergency contraception (the “morning after pill”) was least available, being in stock for only 31% of the observation period (Table 1).
  Stock-outs of contraceptives were both rare and short-lived. All methods except implants were stocked out for less than 3% of months observed. Implants were stocked-out for 11% of months. On average, contraceptive materials were stocked out less than one day per month (Tables 2 and 3).
B. **Despite availability, utilization of family planning services was low.**
  Although contraception was generally available, utilization of family planning services by women in the study area was low. Rates of utilization ranged from 0.6% to 6.1%, averaging 2.96% of women overall.
  In univariate linear regression, utilization demonstrated a positive relationship to: (a) the number of different types of contraceptives available in a clinic (Fig. 3), (b) availability of trained providers to administer the various methods, and (c) engagement of providers in offering family planning services (Fig. 4). There was no significant association between uptake ratio and number of contraceptives stocked out (Table 4). In a multivariate analysis including all of these variables, the number of health professionals engaged and the number of types of contraceptives available were found to be statistically significant in increasing the uptake ratio (*p* < 0.001) (Table 4).
C. **What explains low uptake of available contraceptive methods in this rural Burundian setting?**
  Analysis of survey and qualitative data from ten in-depth interviews with facility managers as well as community leaders and seven FGDs 6–7 participants each of women that were pregnant, not pregnant, and men (*n* = 47) revealed four factors likely contributing to low uptake of contraceptives in this rural Burundian setting, as follows.
  1. **Lack of providers to administer contraceptive methods**
    Lack of providers is a first contributing factor. On average at these health facilities, 5.6 providers served populations of more than 9000 individuals. Only about one-fifth (22.4%) were trained to administer the full array of family planning methods.
  2. **Lack of fit between available and preferred contraceptive methods**
    The least available long-term method of contraception was the most preferred by women. Implants were the long-term contraceptive method least plentiful at participating facilities, available only about 40% of the time. Yet when women sought family planning services, the method they were the most interested in was implants. The following citations from the qualitative data illustrate:
*“Many women like implants.”*
*-Female health facility manager, individual interview*
    “*Women are happy with implants. It’s not the case for pills. The health providers keep on shifting appointments. Women appreciate implants because they can spend three years without any need to go back to the provider.”*
*-Female, five children, FGD*
  3. **A “Climate of Fear”**
    The “climate of fear” that seemed to surround contraceptive use in these communities also sheds light on uptake. A recurring theme in FGDs with women about contraception was “fear.” As one woman put it:
*“We are scared. When you go [for family planning]*, *you don’t tell anybody.”*
    -*Female*, *four children*, *FGD*
    What are women afraid of? Disclosure of use of family planning is a major fear for women. They are ashamed and afraid of being seen by others when seeking family planning services.
    Contraception is condemned by the leaders of conservative Christian churches in the area, who refer to it as a means of killing an unborn person that will result in eternal punishment. The following quote illustrates:
*“There were meetings priests were conducting*, *what we call recollections*, *and all they were doing was to oppose the use of contraceptives. They even reached the point of saying: ‘If you decide to do this*, *the consequence will be death. You will all spend eternity in hell because you buried in you all the dead bodies of the children you were supposed to give birth to.’”*
    -*Male*, *six children*, *FGD*
    The harsh terms in which opposition to contraception is put results in women fearing for their personal safety when seeking contraceptive services.
*“You cannot even* [*greet someone at clinic*] *or say you know him/her. Do you understand? There are some members of a church who are opposed to that* [*contraceptive use*]. *If they hear that I use them*, *I will have problems. For my security*, *let me go secretly. This is a reality*.”
    -*Female*, *pregnant*, *FGD*
    The teachings of religious authorities have a significant impact on potential contraceptive users, health providers, and the larger community. In the absence of easy communication with the outside world (radio, TV, internet), many accept these teachings as “truth.”
*“Many women obeyed those teachings of priests. Even those who were using them decided to stop. All those who were using them have quit.”*
    -*Male*, *six children*, *FGD*
    Women who use contraception are defying church leaders. They risk social isolation and rejection by friends and neighbors if their actions become known to others. Social isolation brings real risks in poor rural societies, where being on good terms with others is essential for leveraging the help and support needed to cope with continuing poverty. A health facility manager acknowledged the isolating consequences of disclosure of contraceptive use when he said:
    “[*A woman using contraceptives*] *needs to hide*, *because if the church leaders catch her*, *they can exclude her. During social events*, *she will not have anybody from her church to support her. It is clear that churches forbid their adherents to use these methods.”*
    -*Male health facility manager*, *individual interview*
    Women also fear disclosure from breach of patient confidentiality by health care providers. Many providers are church followers and disapprove of contraception themselves. As a result, women fear staff will report their use of contraception. This is especially true when a provider attends the same church and is involved in church activities. A provider explains:
*“… when they come and don’t find me here*, *they fear to be received by my colleagues who are their church mates. They fear that they may talk about it.”*
    -*Female health facility manager*, *individual interview*
    Lack of private space in which to receive family planning clients at health facilities also leads to fear of disclosure. Most facilities do not have separate waiting areas for women seeking contraception. They wait to be seen with the other patients, many of whom are clearly sick. Not being sick themselves, these women stand out. They worry others will guess the purpose of their visit. In the following quote, a provider explains how health facility layout causes some women to abandon appointments for family planning services:
*“The major causes* [*of low contraceptive use*] *are related to how this health center is built. Patients and people coming for contraception services meet in one place. Clients coming for family planning methods don*’*t appreciate it. There are some who prefer to abandon* [*seeking family planning services*] *and others who accept to wait. This is why the number is low.”*
    -*Male health facility manager*, *individual interview*
    Women using contraceptives also fear conflicts with disapproving spouses. The stakes of such conflicts may be especially high if the woman depends on her husband for financial support. Major conflicts may result in withdrawal of such support – a real threat for economically dependent wives. To avoid this, women may choose to hide their use of contraceptives from their husbands, as in the following case:
*“I never told my husband. It was a secret between a nurse working in this health center and me. One day he saw pills I had put somewhere and asked me: ‘Where did you get these pills from?*’ *and I replied that I don*’*t know. I never told him. I was the one to get tired* [*of closely spaced pregnancies*]. *It was my problem; I didn*’*t tell him*.”
    -*Female community leader*, *nine children*, *individual interview*
    People in these rural Burundian communities are not well informed about modern contraceptives. Lack of knowledge also causes fear. Women fear reports they hear – rumors circulating in the villages – that contraceptives cause sterility and cancer – a fatal disease, since no treatments are available. Women also fear side effects, such as heavy bleeding, headache, menstruation problems, and generally feeling ill. Women who have neither used family planning themselves nor sought information from knowledgeable professionals rely on information from others. When this information is negative or vague, it leaves the hearer feeling hesitant. We see this happening here:
*“When you look at it*, *family planning is good. But when you listen to someone who has used them*, [*and*] *she tells you: ‘It happened to me like this or like that*.’ *And another one tells you: ‘Things are like this*.’ *You become terrified. And you think: ‘And if they cause me some trouble*, *what would I do?*’ *And you hesitate…”*
    -*Female*, *pregnant*, *FGD*
*“Even if you think of using them*, *when you already know what happened to such and such*, *I think you cannot just fall in. For instance*, *it is said that these injectables cause tumors. They also say that using them causes menstruations to stop or bleeding keeps on coming until*, *until*, *until… You can even get pregnant while using these methods.”*
    -*Female*, *pregnant*, *FGD*
  4. **Refusal of Family Planning Services by Providers**
    A fourth contributor to low uptake of contraceptives is refusal of services by providers. Providers who are also church followers may refuse family planning services to women, citing inconsistency with religious beliefs. Women who are refused services spread word of their experience to other women, and demand drops. One provider described this as follows:
*“Among us here*, *the staff*, *there is a problem. We are five* [*nurses*] *but three of us refuse to receive people who need these services because of the church teachings. …They send away clients who come for these services*, *and they go back home*, *without receiving the methods. They tell others that this nurse or that one does not receive people who need contraceptives at that facility. This is a big challenge we face here*.”
    -*Male health facility manager*, *individual interview*

**Table 1:** Availability of contraceptive materials

Type of contraceptive	% (*n* = 427)
Long term methods	
Depot medroxyprogesterone acetate [DMPA]	99.8
Implant	40.5
Intra-uterine device [IUD]	82.7
Short term methods	
Oral contraceptives	98.8
Condom (male)	97.4
Condom (female)	54.8
Emergency contraception (Morning after pill)	31.1

**Table 2:** Stock-outs for available contraceptive methods across 39 health centers

Type of contraceptive	% (*n* = 427)*
Long term methods	
Depot medroxyprogesterone acetate [DMPA] (*n* = 426)	0.9
Implant (*n* = 173)	10.9
IUD (*n* = 353)	1.1
Short term methods	
Oral contraceptives (*n* = 422)	1.7
Condom (male) (*n* = 416)	1.4
Condom (female) (*n* = 333)	2.6
Emergency contraception (Morning after pill) (*n* = 133)	0

*Sample size is less than 427 since some contraceptive methods were not available at all of the facilities

**Table 3:** Average number of days per month of stock outs for the available contraceptives

Type of contraceptive	Mean	SD	Min	Max	Median	25% Interquartile Range	75% Interquartile Range
Long term methods							
Depot medroxyprogesterone acetate [DMPA] (*n* = 426)	0.03	0.4	0	6	0	0	2
Implant (*n* = 173)	0.9	4.3	0	29	0	0	20
Intra-uterine device [IUD] (*n* = 353)	0.2	2.3	0	30	0	0	6
Short term methods							
Oral contraceptives (*n* = 422)	0.1	1.5	0	23	0	0	6
Condom (male) (*n* = 416)	0.1	1.6	0	30	0	0	1
Condom (female) (*n* = 233)	0.5	3.8	0	31	0	0	25
Emergency contraception (Morning after pill) (*n* = 133)	0	0	0	0	0	0	0

**Table 4:** Multivariate linear regression of uptake ratio onto number of health professionals engaged in family planning services provision, number of health professionals fully trained in family planning service provision, types of contraceptives available and number of contraceptives stocked out (*n* = 427)

	Univariate analysis	Multivariate analysis*
	Beta (95% CI)	Beta (95% CI)
	*p*-value	*p*-value
Number of health professionals engaged	0.0040341 (0.0028246, 0.0052435)	0.002981 (0.0016179, 0.0043441)
	<0.001	<0.001
Number of health professionals trained	0.0032803 (0.000626, 0.0059347)	−0.0028119 (−0.0058121, 0.0001884)
	0.016	0.066
Number of types of available contraceptives	0.0045371 (0.0030997, 0.0059746)	0.0037303 (0.0019481, 0.0055125)
	<0.001	<0.001
Contraceptives stocked out	−0.0044289 (−0.0110464, 0.0021886)	−0.0028839 (−0.0068344, 0.0010665)
	0.187	0.152

*For multivariate analysis, all four predictor variables were included in the model simultaneously

## Discussion

This mixed-methods study assessed uptake of modern contraceptives and barriers to contraceptive use among women of reproductive age (15–49 years) in two health districts of rural Burundi. Though contraceptives were generally available in the facilities studied, with at least one long term and one short term method available 99% of the time, utilization was very low. Only about 3% of women of reproductive age sought family planning services over the 12-month study period. In univariate linear regression, utilization varied in direct relationship to: (a) the number of different types of contraceptives available in a clinic, (b) availability of trained providers to administer the various methods, and (c) engagement of providers in offering family planning services. In a multivariate analysis including all of these variables, the number of health professionals engaged and the number of types of contraceptives available were found to be statistically significant in increasing the uptake ratio.

To understand the low contraceptive use despite the availability of contraceptive methods, a qualitative study involving individual interviews with facility managers and community leaders as well as focus group discussions with pregnant women, non-pregnant women, and men was carried out. Four barriers to contraceptive use identified through the research help to explain the low utilization rate. First is a lack of providers fully trained to administer contraceptive methods. Second is a lack of fit between the methods preferred by women and those most easily available. Third, a “climate of fear” surrounds contraceptive use for women. Finally, some providers refuse to provide family planning services.

**Fig. 5:**
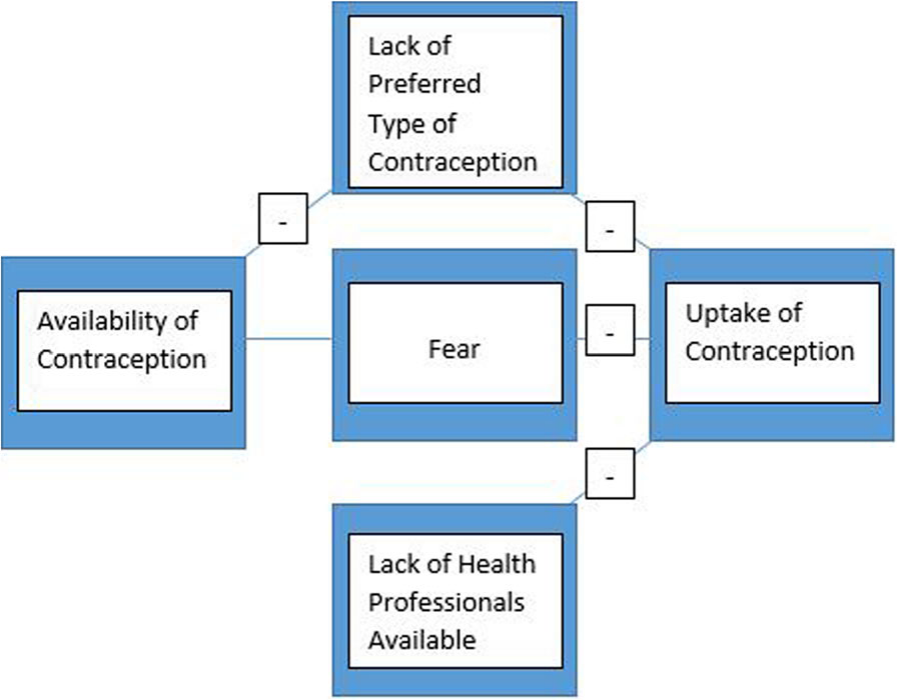
Conceptual framework for factors inhibiting uptake of contraceptive services

A conceptual framework linking quantitative and qualitative findings is presented in Fig. 5. The quantitative results demonstrated that although there was adequate availability of contraceptives, uptake remained low. Qualitative and quantitative findings indicated that several factors may have reduced uptake, including limited availability of preferred contraceptives, lack of health professionals available to offer family planning, and the fear of accessing contraceptive services.

The “climate of fear” surrounding contraceptive use in this rural Burundian region appears at least partially rooted in conservative Christian religious doctrine condemning family planning. A small, related body of research examining the relationship of religion to family planning uptake in African countries has yielded mixed results. A Zambian study showed religious beliefs accounted for 50% of decisions to decline the use of contraceptives [27], whereas other studies have indicated that church affiliation does not always prevent contraceptive use [28, 29]. An analysis of religious involvement in Mozambique revealed a net positive association between church attendance and use of contraceptives regardless of denominational affiliation [30].

Rural Burundi’s extreme poverty must also be considered in accounting for low uptake of family planning in the region. Our findings reflect a scarcity of resources at both state and individual levels. State-level poverty is manifested in the infrastructure of health care. Few health clinics serve a large rural area in the study setting, and these clinics are small and lack private space for provision of family planning services. Availability of preferred contraceptive methods at these clinics is poor, and transportation is inadequate. State-level poverty is also manifested in the lack of well-trained health professionals [31] to provide family planning services, treat side effects, and provide educational services.

Individual-level poverty is evident in low levels of education, and consequent low literacy. Illiteracy prevents individuals from accessing objective information on family planning. Many Burundians are subsistence farmers with little outside income. They lack material assets (radios, cell phones, internet access) that could provide links to information beyond their local communities [32, 33].

Such “asset poverty” helps to explain the influence of religious teachings and rumors on ideas and feelings about contraceptive use. Without other options for information-gathering, people must rely on church leaders and neighbors.

Community-based distribution (CBD) of family planning, in which services are delivered outside health care settings by community health workers (CHWs), has long been recognized as an effective means of improving access to family planning in remote locations [34, 35]. CBD has considerable potential to address uptake barriers associated with clinic-based services, such as transportation difficulties, lack of privacy, and fear of disclosure of contraceptive use and subsequent stigmatization [36]. In the future, community-based distribution may usefully expand to include self-administered and/or home-based services [37]. Other interventions demonstrated to improve uptake include male involvement in family planning and improved communication in couples [38, 39]. A critical, yet understudied area is how best to provide information on contraception and sexual and reproductive health information for rural youth in resource-limited settings.

This study has a number of limitations that should be pointed out. First, the qualitative component was carried out in two purposefully sampled health districts of Burundi; results are not generalizable. No quantitative data were collected on women’s use of particular contraceptive methods. Data on attendance at contraceptive education sessions could not be captured; thus we were unable to examine the relationship between attendance at these sessions and contraceptive uptake. Future studies should assess uptake by method and compare what women prefer most to what health professionals recommend. It is also important to understand how availability of specific methods such as implants affects women’s satisfaction with the family planning services.

## Conclusions

In resource-limited, rural locations in Africa, where access to information is scarce, availability of modern family planning methods alone will likely not ensure uptake, especially when access is limited to clinical settings. Multi-pronged intervention efforts that work to address multiple uptake barriers simultaneously have the greatest chance of success. In the rural Burundian setting, these efforts might include campaigns to provide accurate information on family planning to the general public; community distribution of contraceptive methods, especially those most preferred by women; training for health professionals and community health workers that includes education on the economic and health benefits of family planning as well as skills for safe and effective administration of contraceptive methods; and strengthening of the health infrastructure.

## Additional files

Additional file 1: Health facility survey instrument. (DOCX 21 kb)
Additional file 2: Qualitative interview guide for women. (DOCX 16 kb)
Additional file 3: Qualitative interview guide for men. (DOCX 15 kb)
Additional file 4: Qualitative interview guide for health facility managers. (DOCX 13 kb)

## Abbreviations

CBD: Community-based distribution
CHW: Community health worker
DMPA: Depot medroxyprogesterone acetate
FDG: Focus group discussion
IUD: Intra-uterine device
