# Supplementary material for: Understanding low uptake of contraceptives in resource-limited settings: a mixed-methods study in rural Burundi
Source: BMC Health Serv Res. 2017 Mar 15;17:209. doi: 10.1186/s12913-017-2144-0 (PMC5353936; doi:10.1186/s12913-017-2144-0)
Supplement: Additional file 1: — Health facility survey instrument. (DOCX 21 kb) [file 12913_2017_2144_MOESM1_ESM.docx]

**Appendix A:**

**FAMILY PLANNING IN RURAL BURUNDI: ASSESSEMENT OF BARRIERS OF CONTRACEPTIVE USE**

**District hospital:………………………………………………or Health center………………………………………………**

**Month/Year:_____________________**

**Date of data collection ________________ (dd/mm/yyyy)**

1. Availability of modern contraceptives at the health facility level for this month.
   1. Pills yes □ No □ DK □
   2. Depo provera Yes □ No □ DK □
   3. Implants Yes □ No □ DK □
   4. Condom-male yes □ No □ DK □
   5. Condom-female yes □ No □ DK □
   6. Intrauterine Device or IUD yes □ No □ DK □
   7. Other………………………………… yes □ No □ DK □
2. Stock-outs of contraceptives at the health facilities for this month:
   1. Pills yes □, how many days…………… No □ DK □
   2. Depo provera Yes □ how many days…………… No □ DK □
   3. Implants Yes □ how many days…………… No □ DK □
   4. Condom-male yes □ how many days…………….. No □ DK □
   5. Condom-female yes □ how many days……………… No □ DK □
   6. Intrauterine Device or IUD yes □ how many days……………... No □ DK □
   7. Other………………………………. yes □ how many days……………... No □ DK □
3. What is the number of people in this hospital’s or health center’s catchment area (for this month): ……………..
   1. (3a) Number of women 15-49 years of age (for this month): ……………
   2. (3b) Number of men 15-49 years of age (for this month): ……………..
4. What is the number of women who came for the family planning program in this hospital or health center for this month? …………………………………

5. Technical competence

a) How many health professionals in the health facility (average for this month): …………………

b) How many of these are trained in providing family planning services (average for this month):………

c) How many staff are engaged in family planning services (average for this month):………............

6 . When family planning services are available (for this month)?

Total number of days during this month family planning services are available: ………….

Total number of hours during this month family planning services are available: ………….

Are family planning services available during the weekend? yes □ No □ DK □

Are family planning services available during the evenings? yes □ No □ DK □

7. Does your facility offer education on family planning (for this month)? yes □ No □ DK □

- 1. (7a) Family planning education for females (for this month): yes □ No □ DK □
  2. (7b) Family planning education for males (for this month): yes □ No □ DK □

8. If yes, by whom? ………Male…………Female…………….and how often? □ (1) More than once a week

□ (2) Once a week

□ (3) Several times a month

□ (4) Once a month

□ (8) Don’t know

□ (9) Not applicable
