# Supplementary material for: Understanding low uptake of contraceptives in resource-limited settings: a mixed-methods study in rural Burundi
Source: BMC Health Serv Res. 2017 Mar 15;17:209. doi: 10.1186/s12913-017-2144-0 (PMC5353936; doi:10.1186/s12913-017-2144-0)
Supplement: Additional file 2: — Qualitative interview guide for women. (DOCX 16 kb) [file 12913_2017_2144_MOESM2_ESM.docx]

**Appendix F: Interview guide with women (pregnant and non-pregnant)**

**Assessment of the Barriers of Contraceptive Use through Qualitative Research.**

Research questions: *what are the main barriers of contraceptive use in rural Burundi?*

This interview guide will focus on people’s beliefs on contraception and contraceptive use, relationship between traditional beliefs about family size and religious beliefs on contraceptive behavior. It aims also to describe how economic dependence affects contraceptive use for women and the potential effect t of the recent civil war on contraceptive use.

I want first to thank you for taking time to speak with me today. My name is (nurse TBA) and I would like to talk to you about family planning. Specifically, I want to understand the main barriers of contraceptive use here in this region. Whatever you tell me will be confidential. This means that I won’t share your responses to anyone else and I will ensure that any information I include in my report does not identify you as interviewee. Maintaining confidentiality is a requirement of group participation and we ask that you do not share what is discussed with those outside of the group or among each other after the end of the focus group. Please feel free to share with me your thoughts and beliefs on contraceptive use.

1. You attended to antenatal care clinic today, how was your visit?

2. How did you decide to get pregnant this time?

For non-pregnant women: How did you last time decide to get pregnant? What was your experience last time you were seeking for antenatal care clinic? Do you plan to have another baby?

3. How many children do you have? How many would like to have? What is xx children your ideal family size? ( probes, how does that related to your religion? How many siblings do you have? How many siblings does your husband have? How does that influence your ideal family size?

4. What do you know about modern contraceptives?

a) Would you give me some examples of contraceptive methods that you know?

5. What are your thoughts about using contraceptives? (General probes: Tell me about that)

Do you know anyone who uses family planning methods? What contraceptive method do people prefer most? Why?

Tell me about a story about what you know about that person using contraception.

Specific probes:

a) In what ways does a husband affect his wife’s thoughts about contraception? What would happen to you if you have a different viewpoint to your husband on using contraceptive methods? (Probe to get a specific example) b) What does your religion say about contraception?

c) Is there anyone here who would be willing to share a story about her personal experience with contraception?

6. Do you know anybody who tried contraception and failed to adhere? What happened to her?

7. Do you know of anybody who wanted to obtain or tried contraception but was unable to access the care that she needed? Why did that occur? 8. My last question is about the impact of the recent civil war which lasted for more than 13 years in Burundi. How the recent civil war did influence your decision of your desired number of children? In what ways? How does this related to your decision of contraceptive use?

9. What do you think shall be done to improve family planning services in this region?

Do you have any question for me?

Thank you very much for your time.
