# Supplementary material for: Understanding low uptake of contraceptives in resource-limited settings: a mixed-methods study in rural Burundi
Source: BMC Health Serv Res. 2017 Mar 15;17:209. doi: 10.1186/s12913-017-2144-0 (PMC5353936; doi:10.1186/s12913-017-2144-0)
Supplement: Additional file 4: — Qualitative interview guide for health facility managers. (DOCX 13 kb) [file 12913_2017_2144_MOESM4_ESM.docx]

**Appendix H: Interview guide with facility managers**

Introduction:

I want first to thank you for taking time to speak with me today. My name is Melino Ndayzigiye and I would like to talk to you about family planning services. Specifically, I want to understand the main barriers of contraceptive use here in this region. In this interview we will discuss your views of the how contraception is used and/or not used by patients.

Whatever you tell me will be confidential. This means that I won’t share your responses to anyone else and I will ensure that any information I include in my report does not identify you as interviewee.

Please feel free to share with me your views of how contraception is used and/or not here.

1. How long have you been working in this area? How long have you been managing this health facility?

2. How would you describe the uptake of contraceptive methods in your facility?

3. What contraceptive methods are available to your patients? Are some more available than others? Why is that?

4. What do you think are the main reasons for [low/high] uptake by patients in your facility? (probes: tell me more about that. In what ways? Etc.)

5. What are the mains challenges you experience in your work providing family planning services?

6. How often do you experience stock-outs of contraceptives? What are the main reasons for those stock-outs?

7. What do you think should be done to increase the uptake of contraceptives in your facility? (Probe to elicit multiple responses, then probe each)

8. What else should I know in order to understand the uptake of family planning services here?

Is there anything more to add to what you’ve told me?

Do you have any question for me?

Thank you very much for your time.
